# Comparison of [HSO_4_]^−^, [Cl]^−^ and [MeCO_2_]^−^ as anions in pretreatment of aspen and spruce with imidazolium-based ionic liquids

**DOI:** 10.1186/s12896-017-0403-0

**Published:** 2017-11-15

**Authors:** Zhao Wang, John Gräsvik, Leif J. Jönsson, Sandra Winestrand

**Affiliations:** 1 Department of Chemistry, Umeå University, SE-901 87 Umeå, Sweden; 2 Present address: Iggesund Paperboard, SE-825 80 Iggesund, Sweden

**Keywords:** Hardwood, Softwood, Pretreatment, Ionic liquid, Enzymatic saccharification, Torrefaction, Xylan, Klason lignin, Pseudo-lignin

## Abstract

**Background:**

Ionic liquids (ILs) draw attention as green solvents for pretreatment of lignocellulose before enzymatic saccharification. Imidazolium-based ILs with different anionic constituents ([HSO_4_]^−^, [Cl]^−^, [MeCO_2_]^−^) were compared with regard to pretreatment of wood from aspen and spruce. The objective was to elucidate how the choice of anionic constituent affected the suitability of using the IL for pretreatment of hardwood, such as aspen, and softwood, such as spruce. The investigation covered a thorough analysis of the mass balance of the IL pretreatments, the effects of pretreatment on the cell wall structure as assessed by fluorescence microscopy, and the effects of pretreatment on the susceptibility to enzymatic saccharification. Torrefied aspen and spruce were included in the comparison for assessing how shifting contents of hemicelluloses and Klason lignin affected the susceptibility of the wood to IL pretreatment and enzymatic saccharification.

**Results:**

The glucose yield after IL pretreatment increased in the order [Cl]^−^ < [HSO_4_]^−^ < [MeCO_2_]^−^ for aspen, but in the order [HSO_4_]^−^ < [Cl]^−^ < [MeCO_2_]^−^ for spruce. For both aspen and spruce, removal of hemicelluloses and lignin increased in the order [Cl]^−^ < [MeCO_2_]^−^ < [HSO_4_]^−^. Fluorescence microscopy indicated increasingly disordered cell wall structure following the order [HSO_4_]^−^ < [Cl]^−^ < [MeCO_2_]^−^. Torrefaction of aspen converted xylan to pseudo-lignin and changed the glucose yield order to [HSO_4_]^−^ < [Cl]^−^ < [MeCO_2_]^−^.

**Conclusions:**

The acidity of [HSO_4_]^−^ caused extensive hydrolysis of xylan, which facilitated pretreatment of xylan-rich hardwood. Apart from that, the degree of removal of hemicelluloses and lignin did not correspond well with the improvement of the enzymatic saccharification. Taken together, the saccharification results were found to mainly reflect (*i*) the different capacities of the ILs to disorder the cell wall structure, (*ii*) the recalcitrance caused by high xylan content, and (*iii*) the capacity of the [HSO_4_]^−^-based IL to hydrolyze xylan.

**Electronic supplementary material:**

The online version of this article (10.1186/s12896-017-0403-0) contains supplementary material, which is available to authorized users.

## Background

Increased energy demand, the use of unsustainable fossil resources, and environmental issues contribute to making renewable resources into attractive feedstocks for fuels and chemicals. Lignocellulosic residues from agriculture and forestry serve as an abundant non-food renewable resource that has great potential to contribute to the supply of fuels, chemicals, materials, and livestock feed [1, 2].

Cellulose and hemicelluloses in lignocellulosic materials can be converted to ethanol and other commodities through saccharification and fermentation. Pretreatment is required to overcome the recalcitrance of lignocellulosic materials to bioconversion [3]. Ionic liquids (ILs) are non-volatile and green solvents that can dissolve lignocellulosic materials under mild conditions and which are therefore interesting to use for pretreatment [3–5]. Cellulose regenerated by using anti-solvent exhibits a more amorphous structure and is easier to degrade using cellulases [6, 7]. Imidazolium-based ILs are well studied with respect to pretreatment of lignocellulosic biomass [4, 8]. The capability of ILs to dissolve or partly dissolve lignocellulose has been strongly, albeit not exclusively, linked to the functionality of the anion [4, 9]. Two approaches for pretreatment of lignocellulose with ILs have been proposed, viz. the Dissolution Process and the Ionosolv Process [4]. A Dissolution Process is based on an IL with a basic anion, such as [MeCO_2_]^−^, whereas an Ionosolv Process is based on an IL with an acidic anion, such as [HSO_4_]^−^. The Dissolution Process involves complete dissolution of the lignocellulosic feedstock. This is possible through the use of ILs with high basicity to which the anion is the dominant contributor [4]. It has been shown that there is a clear correlation between the basicity of the anion and the capability of the IL to dissolve cellulose [4]. The dissolving power of ILs with basic anions has been linked to strong interactions between the anion and the hydroxyl groups of the cellulose [4]. The Ionosolv Process would only partially dissolve the lignocellulosic feedstock. The lignin and the hemicellulose would be partially dissolved, while the cellulose would remain intact. Lignin dissolution is done through acid-catalyzed hydrolysis of the β-O-4 linkage in lignin [9]. Water and protic ILs with acidic anions, such as [HSO_4_]^−^, are sufficient to make that work [9]. The IL, the acidity, and the water all play a role in the effectiveness of the cleavage of ether bonds in lignin [9]. Apart from the functionality, the cost of the ILs remains an issue [3, 5, 7]. Recycling [5, 10, 11] and using less expensive ILs [5] may be possible ways to make IL pretreatment affordable. Recycling and decreasing the cost of the IL would be easier in an Ionosolv Process due to better tolerance to moisture and better IL stability [4, 5, 12]. Therefore, acidic ILs, such as [HSO_4_]^−^, would have advantages with regard to large-scale utilization.

A previous study [13] on four imidazolium-based ILs ([C = C_2_C_1_im][MeCO_2_], [C_4_C_1_im][MeCO_2_], [C_4_C_1_im][Cl], and [C_4_C_1_im][HSO_4_]) showed that they exhibited different efficiency when used for pretreatment of a hardwood substrate (hybrid aspen) and a softwood substrate (Norway spruce). For both the hardwood and the softwood, the acetate-based ILs ([C_4_C_1_im][MeCO_2_] and [C = C_2_C_1_im][MeCO_2_]) were most efficient with regard to enhancing the glucose yield after enzymatic saccharification of IL-pretreated wood. For pretreatment of hybrid aspen, [C_4_C_1_im][HSO_4_] was far superior to [C_4_C_1_im][Cl] resulting in more than twice as high sugar yields, and it was almost as efficient as the two acetate-based ILs [13]. For pretreatment of Norway spruce, [C_4_C_1_im][Cl] gave more than twice as high sugar yields as [C_4_C_1_im][HSO_4_] [13]. However, as the chemical effects of the IL pretreatment and the mass balance were not studied [13], the discrepancy between the effects of the ionic liquid on hardwood and softwood was not explained. This work is an extension of the previous study [13] and is focused on elucidating the reasons behind different effects on hardwood and softwood through performing a thorough mass balance analysis and through the study of structural as well as chemical effects. This in-depth study also includes more refined fractions of the woody materials than the previous study. ILs that could be used in both Ionosolv and Dissolution processes [4] are covered by the experiments, whereas recycling was considered to fall outside the scope of the investigation.

Softwood, such as spruce, contains more lignin than hardwood, such as aspen, something that may impede enzymatic saccharification of cellulose [3, 14]. Brandt et al. [4] concluded that while hydrogen-sulfate-containing ILs, such as [C_4_C_1_im][HSO_4_], had shown even better delignification effect than corresponding acetate-based ILs, the delignification effect of chloride-based IL was comparatively low. Karatzos et al. [8] found that the pretreatment efficiency of ILs was related to their ability to dissolve the feedstock, and reported the composition of the material before and after pretreatment as well as the mass balance. However, as only one species of biomass, sugarcane bagasse, was included in that investigation [8], the different effects of ionic liquids on hardwood and softwood remain to be fully elucidated.

For the reasons stated above, we compared the composition and mass balance of aspen and spruce after pretreatment with ILs based on different anions, viz. [C_4_C_1_im][HSO4], [C_4_C_1_im][Cl], and [C_4_C_1_im][MeCO_2_]. To further analyze the impact of the ILs on the constituents of the wood, torrefied aspen and spruce were included in the investigation. Torrefaction typically results in decomposition of hemicelluloses and formation of pseudo-lignin [15], a Klason-lignin-positive aromatic polymeric substance derived from carbohydrate [16]. Thus, for torrefied wood the influence of hemicellulose has been decreased and the influence of Klason lignin has been increased. Apart from being included in the investigation for analytical reasons, torrefaction is also of interest with regard to efficient transport and handling of lignocellulosic biomass [17, 18].

## Methods

### Preparation of wood samples

The biomass samples used in this study were extractive-free wood of hybrid aspen and Norway spruce, and the corresponding torrefied materials, hereafter referred to as T-aspen and T-spruce. The aspenwood and the spruce wood were milled with an A11 Basic Mill (IKA, Staufen, Germany) and sieved with 100–500 μm sieves (Retsch Analytical AS 200, Retsch, Haan, Germany). The milled and sieved aspenwood and spruce wood were freeze-dried to a dry-matter content of 100%. The moisture content was measured using an HG63 Moisture Analyzer (Mettler-Toledo, Greifensee, Switzerland). Using a previously described procedure [19], portions of 3 g of freeze-dried wood of aspen or spruce were extracted with 200 mL of a 9:1 mixture of petroleum ether (Petroleum Benzene, Merck, Darmstadt, Germany) and acetone by performing 15 extraction cycles with a Soxhlet system (Büchi Extraction System B-811, Büchi, Flawil, Switzerland) followed by air-drying for ~16 h.

Portions of extractive-free wood of aspen and spruce were torrefied in a torrefaction reactor with nitrogen gas at 285 °C for 16.5 min. The weight of the extracts and the losses during the torrefaction were measured. The fraction of extractives amounted to (% dry weight): 0.90 ± 0.16 for aspen and 1.07 ± 0.03 for spruce, whereas the mass remaining after torrefaction (% dry weight) was 57.9 ± 2.7 for aspen and 73.3 ± 4.2 for spruce.

### Ionic liquids and Karl-Fischer titration

The three ILs [C_4_C_1_im][HSO_4_], [C_4_C_1_im][Cl], and [C_4_C_1_im][MeCO_2_] (all of which are based on the 1-butyl-3-methylimidazolium cation, a.k.a. [Bmim]) were prepared as previously described [13]. The ILs were dried overnight at 65 °C under stirring using an evaporator and a high vacuum pump. In accordance with Brandt et al. [20], the water content of [C_4_C_1_im] [HSO_4_] was adjusted to 20% by addition of ultra-pure water (Millipore, Billerica, MA). The water content of the other two ILs was <1% as determined by Karl-Fischer titration.

A Karl-Fischer titrator (756 KF coulometer, Metrohm, Herisau, Switzerland) with a diaphragm-free titration cell and Hydranal Coulomat E reagent (Sigma-Aldrich, St. Louis, MO) were used to determine the moisture content of the ILs. After drying, approx. 100 μL of the IL was withdrawn into a desiccator-dried syringe and weighed. After injection of the IL into the Karl-Fischer reactor, the syringe was weighed again to determine the amount of sample injected.

### Ionic liquid pretreatment

The IL pretreatment was performed according to Gräsvik et al. [13] by adding 50 mg wood sample (dry weight) to 950 mg ionic liquid and incubating the reaction at 100 °C for 20 h. The samples were then cooled to room temperature, and the pretreated material was precipitated by addition of 1 g of methanol followed by vigorous mixing. A second precipitation was performed by addition of 10 g of ultra-pure water followed by vigorous mixing. The mixture was centrifuged for 10 min at 14,500 *g* and the supernatant was decanted and collected. The pellet was washed using 3 × 10 g ultra-pure water. After that, the pellet was freeze-dried as a part of the analytical procedure. The washing liquids (~30 g) were collected and mixed with the supernatant (~11 g), and the chemical composition of the resulting liquid (~41 g) was analyzed. In total, four different woody materials were pretreated with three different ILs, resulting in 12 different pellets/liquids. For mass balance analysis, quadruplicate measurements were performed.

### Liquid phase compositional analysis

A portion (0.5 mL) of the liquid phase of each sample was diluted with water (0.5 mL) and the monosaccharide content was analyzed by using high-performance anion-exchange chromatography (HPAEC). Moreover, 14 mL of the liquid phase of each sample were concentrated to 0.25–0.3 mL by freeze-drying. The oligosaccharide content was analyzed by diluting 0.2 mL of the concentrated samples with 0.8 mL of ultra-pure water and acidifying the mixture to a pH of 0.3 with a 72% solution of sulfuric acid (50 μL for samples pretreated with [C_4_C_1_im][HSO_4_] or [C_4_C_1_im][Cl], and 100 μL for samples pretreated with [C_4_C_1_im][MeCO_2_]). The samples were vortexed and autoclaved at 121 °C for 60 min. HPAEC was used to analyze the sugar content, and the oligosaccharide content was calculated by subtracting the original monosaccharide content.

### Solid fraction compositional analysis

The weight of the dried pellet of each IL-pretreated sample was determined to calculate the recovery. The lignin and carbohydrate contents of the pellets and the four woody materials were determined essentially according to Sluiter et al. [21]. Since the woody materials were already extractive-free, no extraction step was included. Fifty mg (dry weight) of each sample were treated with 1.5 mL 72% sulfuric acid at 30 °C in a water bath for 1 h. Samples were diluted to a sulfuric acid content of 4% followed by autoclaving at 121 °C for 1 h. Monosaccharides were determined using HPAEC (section “HPAEC analysis of monosaccharides”) instead of using HPLC. Compositional analysis of lignocellulosic biomass includes a heating step in the presence of sulfuric acid, intended to generate monosaccharides from hemicelluloses and cellulose. Under such conditions there may be some degradation of monosaccharides to furan aldehydes and aliphatic carboxylic acids. Therefore, to make the compositional analysis as thorough as possible, even monosaccharide degradation products were quantitated. HPLC was used to determine the content of furfural, HMF (5-hydroxymethylfurfural), acetic acid, levulinic acid, and formic acid (section “HPLC analysis of furan aldehydes and carboxylic acids”).

### Enzymatic saccharification

IL pretreatment of 50 mg of the woody materials was performed as previously described (section “Ionic liquid pretreatment”). The enzymatic hydrolysis was performed as described by Gräsvik et al. [13]. The pretreated material was washed with 1 × 10 g 50 mM citrate buffer (pH 5.2) and centrifuged for 10 min at 14,500 *g* prior to enzymatic hydrolysis. After washing, 900 mg of 50 mM citrate buffer (pH 5.2) and 50 mg of a 1:1 mixture of Celluclast 1.5 L and Novozyme 188 (liquid enzyme preparations obtained from Sigma-Aldrich, St. Louis, MO, USA) were added to each sample. This would correspond to a loading of ~40 mg protein per g non-pretreated substrate. The reaction mixtures were incubated in an orbital shaker set at 170 rpm and 45 °C (Ecotron incubator shaker, Infors, Bottmingen, Switzerland) for 72 h. The four non-pretreated woody materials were digested in the same way. All reactions were performed in triplicate. Samples (10 μL) were withdrawn from the reaction mixtures at the start and after 2 h of incubation, and were used for analysis of the glucose production rate (GPR) using a glucometer (Accu-Chek Aviva, Roche Diagnostics, Rotkreuz, Switzerland). The glucometer was calibrated with a series of glucose solutions (mM): 1.25, 2.5, 5, 7.5, 10, 12.5, 15, and 17.5. Samples for glucometer analysis were diluted with deionized water and mixed (1:1) with glucometer buffer (0.15 mM sodium chloride, 1.2 mM calcium chloride, 0.9 mM magnesium chloride, 2.7 mM potassium chloride, pH 7.4) to reach the calibration range. The yield of monosaccharides after 72 h was determined using HPAEC (section “HPAEC analysis of monosaccharides”). The monosaccharide yields (g g^−1^ dry woody material) were calculated based on the amount of biomass before pretreatment, which was 50 mg for all samples.

### HPAEC analysis of monosaccharides

HPAEC was used for determination of the contents of arabinose (Ara), galactose (Gal), glucose (Glc), mannose (Man), and xylose (Xyl) in the liquid phase after pretreatment, in compositional analysis of the solid fraction after pretreatment, in compositional analysis of untreated wood, and in enzymatic hydrolysates. All samples were diluted with ultra-pure water and filtered through 0.2 μm nylon membranes (Millipore). The analysis was performed using an ICS-5000 instrument equipped with an electrochemical detector, a CarboPac PA1 (4 × 250 mm) separation column, and a CarboPac PA1 (4 × 50 mm) guard column (all from Dionex, Sunnyvale, CA). The temperature of the column oven was 30 °C. Prior to injection, the column was regenerated with a solution of 260 mM sodium hydroxide (Sodium Hydroxide Solution for IC, Sigma-Aldrich) and 68 mM sodium acetate (Anhydrous Sodium Acetate for IC, Sigma-Aldrich) for 12 min followed by ultra-pure water for 2 min. Each sample was injected once, and elution was performed with ultra-pure water during 25 min. The flow rate was 1 mL min^−1^. External calibration curves were established by using 0.5, 1, 5, 10, 20, and 40 ppm (mg L^−1^) solutions of Ara, Gal, Glc, Man, Xyl [prepared from monosaccharide preparations (>99%) obtained from Sigma-Aldrich].

### HPLC analysis of furan aldehydes and carboxylic acids

Reaction mixtures from the compositional analysis (section “Liquid phase compositional analysis”) were analyzed with regard to carbohydrate by-product formation. Determination of the concentrations of the furan aldehydes furfural and HMF, and the carboxylic acids formic acid, acetic acid, and levulinic acid was performed with an Agilent Technologies 1200 series HPLC system (Agilent, Santa Clara, CA). For analysis of the furan aldehydes, the device was equipped with an autosampler, a diode-array-detector (DAD), a binary pump, and a degasser (all from Agilent), and the chromatographic separation was performed using a 3.0 × 50 mm, 1.8 μm Zorbax RRHT SB-C18 column (Agilent) with aqueous 0.1% (volume fraction) formic acid as Eluent A, and acetonitrile with 0.1% (volume fraction) formic acid as Eluent B. The separation program was: 3 min 3% B, 4 min 10% B, and 2 min post run-time for re-equilibration of the column. The flow rate was set to 0.5 mL min^−1^, the absorption at 282 nm was recorded, and the temperature of the column oven was 40 °C. Quantitation was performed using an external calibration curve covering the interval 5 μM - 250 μM.

For carboxylic acids the device was equipped with an autosampler, a refractive index detector (RID), a binary pump, and a degasser (all from Agilent). The chromatographic separation was performed using a Bio-Rad Aminex HPX-87H column (Bio-Rad Laboratories AB, Solna, Sweden). Separation was achieved with an eluent consisting of an aqueous solution of 0.01 M H_2_SO_4_ applied with a flow rate of 0.6 mL min^−1^. The temperature of the column oven was 60 °C and the temperature of the detector cell of the RID was 55 °C. An external calibration curve covering the interval 5 mM - 250 mM was used for quantitation.

In the mass balance analysis, the masses of formic acid, levulinic acid, and HMF (Additional file 1: Table S1) were converted to glucan by using division factors of 0.28, 0.72, and 0.78, respectively. The mass of furfural (Additional file 1: Table S1) was converted to xylan by using a division factor of 0.73. This was based on the assumption that HMF, levulinic acid, and formic acid were derived mainly from glucan, whereas furfural was derived mainly from xylan.

### Fluorescence microscopy

Fluorescence microscopy was carried out at the Biochemical Imaging Centre of the Chemical-Biological Centre (KBC) (Umeå, Sweden). Following the sample-preparation procedure of Rahikainen et al. [22], the solid residues obtained after the IL treatments were embedded in London Resin White (TAAB Laboratories Equipment Ltd., Aldermaston, England). The specimens were then sectioned (2 μm) with a rotary microtome (Leica RM2245, Germany) and the autofluorescence of lignin was visualized by excitation at 470 nm and emission at 525 nm using a fluorescence microscope (Axioimager Z1, Carl Zeiss MicroImaging GmbH, Göttingen, Germany).

## Results

### Chemical composition of woody materials

The results of the compositional analysis of aspen, T-aspen, spruce, and T-spruce are shown in Table 1. As expected, aspen had a high content of xylan (15.9%) and a low content of total lignin [19.6% Klason lignin and ASL (acid-soluble lignin)]. The acetyl content was as high as 4.6%. Torrefaction resulted in low xylan content (2.6%) and very high Klason lignin content (52.0%). The spruce had a high content of mannan (11.4%), a high content of total lignin (32.5% Klason lignin and ASL), and a low content of acetyl groups (1.1%). Torrefaction of spruce resulted in low hemicellulose content and very high Klason lignin content (47.3%). Thus, for both aspen and spruce torrefaction resulted in a material with low hemicellulose content and high content of Klason lignin and cellulose (Table 1). The high Klason lignin content of the torrefied materials (Table 1) can be attributed to formation of pseudo-lignin.

**Table 1:** Compositional analysis of solids of non-pretreated and IL-pretreated woody samples

Sample	% Dry mass							
	Klason ^a^	ASL ^b^	Arabinan	Galactan	Glucan	Xylan	Mannan	Acetyl
Aspen	12.9 ± 0.2	6.7 ± 0.1	0.7 ± 0.5	1.4 ± 0.1	45.3 ± 1.2	15.9 ± 0.4	2.6 ± 0.1	4.6 ± 0.1
[HSO_4_]	12.9 ± 0.6	3.5 ± 0.3	ND ^c^	ND	62.7 ± 0.6	9.2 ± 0.2	3.4 ± 0.1	1.0 ± 0.1
[Cl]	14.6 ± 0.2	6.8 ± 0.2	ND	1.2 ± 0.1	47.4 ± 0.6	16.5 ± 0.2	2.2 ± 0.1	5.4 ± 0.7
[MeCO_2_]	17.9 ± 0.4	5.8 ± 0.4	ND	0.9 ± 0.1	53.3 ± 1.3	14.0 ± 0.3	2.3 ± 0.1	1.5 ± 0.1
T-aspen	52.0 ± 1.4	2.8 ± 0.1	ND	ND	32.0 ± 0.1	2.6 ± 0.1	1.4 ± 0.1	1.4 ± 0.1
[HSO_4_]	57.7 ± 0.4	1.5 ± 0.1	ND	ND	31.6 ± 1.1	1.4 ± 0.1	ND	ND
[Cl]	53.3 ± 0.5	2.1 ± 0.2	ND	ND	29.0 ± 0.1	2.3 ± 0.1	ND	1.4 ± 0.3
[MeCO_2_]	54.1 ± 0.9	2.6 ± 0.1	ND	ND	30.1 ± 1.2	2.1 ± 0.1	ND	ND
Spruce	28.6 ± 0.8	3.9 ± 0.1	1.0 ± 0.1	2.0 ± 0.1	43.6 ± 0.2	6.0 ± 0.1	11.4 ± 0.1	1.1 ± 0.2
[HSO_4_]	29.9 ± 0.6	3.1 ± 0.1	ND	0.7 ± 0.1	52.5 ± 0.2	3.7 ± 0.1	8.5 ± 0.1	ND
[Cl]	28.4 ± 0.1	4.9 ± 0.1	1.1 ± 0.1	2.0 ± 0.1	43.7 ± 0.9	6.3 ± 0.1	11.1 ± 0.2	0.8 ± 0.1
[MeCO_2_]	26.0 ± 0.6	5.0 ± 0.4	0.8 ± 0.1	1.7 ± 0.1	48.3 ± 0.5	4.9 ± 0.1	11.5 ± 0.0	ND
T-spruce	47.3 ± 0.2	5.9 ± 0.4	ND	0.6 ± 0.1	39.9 ± 0.2	1.6 ± 0.1	3.8 ± 0.1	ND
[HSO_4_]	49.4 ± 1.9	3.6 ± 0.1	ND	ND	43.2 ± 0.1	0.9 ± 0.1	2.0 ± 0.1	ND
[Cl]	43.4 ± 0.4	6.0 ± 1.9	ND	0.6 ± 0.1	40.0 ± 1.1	1.5 ± 0.1	3.2 ± 0.1	ND
[MeCO_2_]	41.8 ± 0.5	5.6 ± 0.2	ND	0.5 ± 0.1	38.9 ± 0.3	1.2 ± 0.0	2.7 ± 0.1	ND

^a^ *Klason* Klason lignin

^b^ *ASL* acid-soluble lignin

^c^ *ND* not detectable

Data based on duplicate measurements

### Effects of pretreatment with ionic liquids

Pretreatment with [C_4_C_1_im][HSO_4_] generally resulted in a decrease of the contents of ASL and hemicelluloses, and an increase or unchanged contents of Klason lignin and cellulose (Table 1). It is noteworthy that the treatment of aspen with [C_4_C_1_im][HSO_4_] resulted in a decrease in xylan content from 15.9% to 9.2%, and a decrease in acetyl content from 4.6% to 1.0%. Pretreatment with [C_4_C_1_im][Cl] resulted in only minor changes in the chemical composition of the four woody samples (Table 1). Pretreatment with [C_4_C_1_im][MeCO_2_] gave different effects for different materials. For aspen, the contents of Klason lignin and cellulose increased, whereas the contents of hemicelluloses decreased. The effects on the composition of T-aspen were small, but the contents of hemicelluloses, which had been reduced by torrefaction, were reduced even further. Pretreatment with [C_4_C_1_im][MeCO_2_] decreased the Klason lignin content for spruce and T-spruce, and in most cases there was also a slight decrease in the contents of hemicelluloses (Table 1).

### Mass balance of ionic liquid pretreatment

The mass balances of the woody materials, before and after pretreatment with the ILs, are shown in Table 2. The data on pretreated materials cover both the solids and the solubilized mono- and oligosaccharides that ended up in the liquid phase (Table 2), and can be used to calculate the fraction of solids remaining after IL pretreatment and the fraction lost for each constituent.

**Table 2:** Mass balance of pretreatment of non-torrefied and torrefied wood of aspen and spruce

Sample		Dry mass (mg)						
		RSM^a^	Arabinan	Galactan	Glucan	Xylan	Mannan	Lignin^b^
Aspen (control)		50.0 ± 0.1	0.16 ± 0.00	0.70 ± 0.01	22.63 ± 0.08	7.96 ± 0.03	1.32 ± 0.01	9.79 ± 0.03
[HSO_4_] Aspen	solid	32.6 ± 0.4	ND^c^	ND	20.44 ± 0.22	3.00 ± 0.04	1.11 ± 0.01	5.36 ± 0.23
	liquid		0.07 ± 0.01	0.37 ± 0.01	0.34 ± 0.01	3.09 ± 0.06	0.15 ± 0.01	
[Cl] Aspen	solid	43.6 ± 0.1	ND	0.50 ± 0.01	20.67 ± 0.09	7.22 ± 0.05	0.98 ± 0.01	9.34 ± 0.12
	liquid		0.04 ± 0.01	0.17 ± 0.02	0.59 ± 0.02	0.04 ± 0.01	0.31 ± 0.02	
[MeCO_2_] Aspen	solid	38.3 ± 0.1	ND	0.34 ± 0.01	20.40 ± 0.30	5.36 ± 0.07	0.88 ± 0.01	9.05 ± 0.24
	liquid		0.06 ± 0.01	0.27 ± 0.02	0.22 ± 0.00	1.58 ± 0.38	0.14 ± 0.02	
T-aspen (control)		50.0 ± 0.1	ND	ND	15.97 ± 0.03	1.32 ± 0.01	0.69 ± 0.01	27.32 ± 0.05
[HSO_4_] T-aspen	solid	44.4 ± 0.6	ND	ND	14.02 ± 0.33	0.63 ± 0.02	ND	26.27 ± 0.35
	liquid		ND	ND	1.12 ± 0.04	0.45 ± 0.02	0.09 ± 0.01	
[Cl] T-aspen	solid	49.3 ± 0.1	ND	ND	14.30 ± 0.11	1.11 ± 0.01	ND	27.34 ± 0.28
	liquid		ND	ND	0.32 ± 0.02	0.09 ± 0.01	0.03 ± 0.01	
[MeCO_2_] T-aspen	solid	47.4 ± 0.6	ND	ND	14.47 ± 0.39	0.99 ± 0.02	ND	26.93 ± 0.49
	liquid		ND	ND	0.61 ± 0.04	0.22 ± 0.02	0.05 ± 0.01	
Spruce (control)		50.0 ± 0.1	0.52 ± 0.01	0.98 ± 0.01	21.85 ± 0.06	3.02 ± 0.01	5.71 ± 0.02	16.27 ± 0.05
[HSO_4_] Spruce	solid	38.7 ± 0.4	ND	0.28 ± 0.01	20.34 ± 0.18	1.45 ± 0.01	3.27 ± 0.03	12.73 ± 0.24
	liquid		0.36 ± 0.05	0.51 ± 0.05	0.41 ± 0.04	0.95 ± 0.09	1.51 ± 0.15	
[Cl] Spruce	solid	45.6 ± 1.1	0.47 ± 0.02	0.92 ± 0.03	19.93 ± 0.41	2.89 ± 0.07	5.07 ± 0.13	15.21 ± 0.36
	liquid		0.03 ± 0.01	0.05 ± 0.01	0.08 ± 0.01	0.01 ± 0.01	0.29 ± 0.03	
[MeCO_2_] Spruce	solid	42.6 ± 0.4	0.37 ± 0.02	0.76 ± 0.01	20.57 ± 0.21	2.09 ± 0.03	4.88 ± 0.04	13.21 ± 0.33
	liquid		0.11 ± 0.01	0.10 ± 0.01	0.06 ± 0.00	0.70 ± 0.11	0.11 ± 0.01	
T-spruce (control)		50.0 ± 0.1	ND	0.29 ± 0.01	19.95 ± 0.09	0.79 ± 0.01	1.90 ± 0.01	26.81 ± 0.12
[HSO_4_] T-spruce	solid	43.9 ± 0.1	ND	ND	18.96 ± 0.04	0.40 ± 0.01	0.89 ± 0.01	23.27 ± 0.67
	liquid		ND	0.15 ± 0.01	0.90 ± 0.06	0.30 ± 0.01	0.86 ± 0.06	
[Cl] T-spruce	solid	48.8 ± 0.8	ND	0.27 ± 0.01	19.45 ± 0.46	0.74 ± 0.02	1.55 ± 0.05	24.11 ± 1.04
	liquid		ND	0.02 ± 0.01	0.47 ± 0.03	0.04 ± 0.01	0.31 ± 0.01	
[MeCO_2_] T-spruce	solid	47.5 ± 0.2	ND	0.22 ± 0.01	18.47 ± 0.11	0.58 ± 0.01	1.28 ± 0.03	22.52 ± 0.28
	liquid		ND	0.03 ± 0.01	0.36 ± 0.02	0.07 ± 0.01	0.21 ± 0.01	

^a^ *RSM* recovered solid mass

^b^ *Lignin* sum of ASL and Klason lignin

^c^ *ND* not detectable

Data based on quadruplicate measurements

The fraction of solids recovered after pretreatment ranged from 65% for aspen pretreated with [C_4_C_1_im][HSO_4_] to 99% for T-aspen pretreated with [C_4_C_1_im][Cl]. For each of the four woody materials, the recovery of solids always decreased in the order [C_4_C_1_im][Cl] > [C_4_C_1_im][MeCO_2_] > [C_4_C_1_im][HSO_4_] (Table 2)_._ For each of the ILs, the recovery of solids was always higher for spruce than for aspen, and always higher for torrefied than for non-torrefied wood. Despite of aspen exhibiting clearly lower recoveries than spruce, the recoveries for T-aspen were similar to those of T-spruce (Table 2).

The fractions of glucan recovered in the solid phase for aspen, T-aspen, spruce, and T-spruce were 90–91%, 88–91%, 91–94%, and 93–97%, respectively. Thus, it was slightly higher for spruce and T-spruce than for aspen and T-aspen. The differences between the ILs were small and did not follow any trend (Table 2).

The recovery of the hemicellulosic polysaccharides arabinan, galactan, xylan, and mannan in the solid phase was generally much lower than that of glucan, but varied depending on wood species and IL (Table 2). Pretreatment with [C_4_C_1_im][HSO_4_] removed about half or more of the xylan in all woody materials, but much of it was recovered as xylose or xylooligosaccharides in the liquid phase (Table 2). For all four materials, the effects on xylan always followed the order [C_4_C_1_im][HSO_4_] > [C_4_C_1_im][MeCO_2_] > [C_4_C_1_im][Cl]. Other hemicellulosic polysaccharides, i.e. mannan, galactan, and arabinan, were also affected by the ILs, typically following the same order. In quantitative terms, the losses of hemicelluloses for aspen (A) and spruce (S) were: [C_4_C_1_im][HSO_4_] 59% (A) and 51% (S); [C_4_C_1_im][Cl] 14% (A) and 9% (S); [C_4_C_1_im][MeCO_2_] 35% (A) and 21% (S). The acidic conditions of the [C_4_C_1_im][HSO_4_] pretreatment can explain its strong effect on hemicellulose. The alkaline conditions of [C_4_C_1_im][MeCO_2_] pretreatment might also contribute to dissolution of hemicelluloses.

The lignin recovery in the solid phase after IL pretreatment was typically >80%, except for [C_4_C_1_im][HSO_4_] pretreatment of aspen and spruce, which resulted in a lignin recovery of 55% and 78%, respectively. Expressed as fraction of lignin lost for aspen (A) and spruce (S), the results were: [C_4_C_1_im][HSO_4_] 45% (A) and 22% (S); [C_4_C_1_im][Cl] 5% (A) and 7% (S); [C_4_C_1_im][MeCO_2_] 8% (A) and 19% (S). Except for T-spruce, the effects on lignin followed the order [C_4_C_1_im][HSO_4_] > [C_4_C_1_im][MeCO_2_] > [C_4_C_1_im][Cl] (Table 2). The effect on T-spruce (84–90% lignin recovery) was larger than the effect on T-aspen (96–100% lignin recovery). The probable explanation for that is that the lignin fraction of T-aspen mainly consisted of pseudo-lignin (the lignin content increased from ~10 mg to ~27 mg after torrefaction) (Table 2), whereas the lignin fraction of T-spruce mainly consisted of real lignin rather than pseudo-lignin (the lignin content increased from ~16 mg to ~27 mg after torrefaction) (Table 2). Thus, the lignin contents of T-aspen and T-spruce were similar, but IL pretreatment seems to have affected real lignin more than it affected pseudo-lignin.

### Fluorescence microscopy

**Fig. 1:**
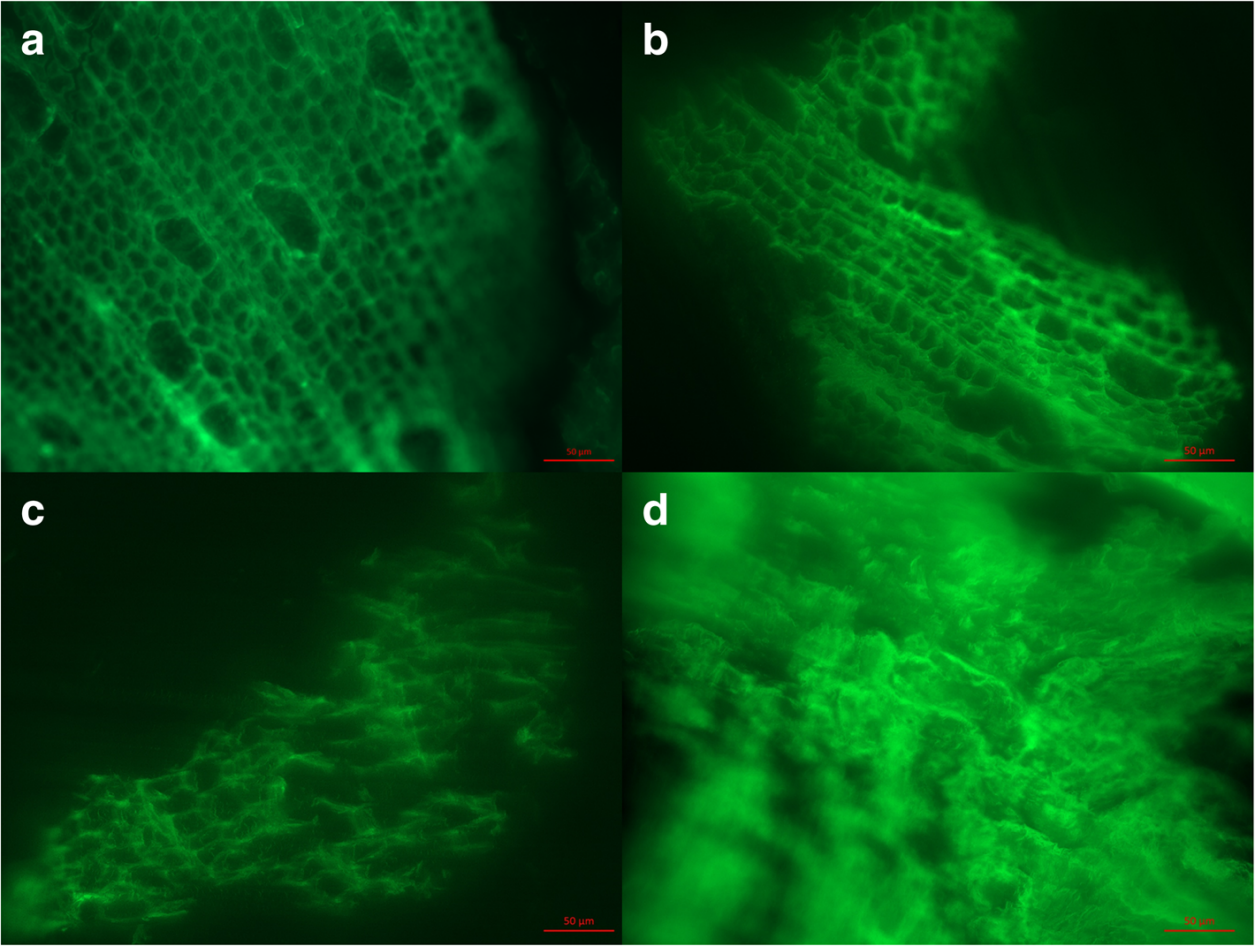
Fluorescence microscopy images of aspen (**a**), [C_4_C_1_im][HSO_4_]-treated aspen (**b**), [C_4_C_1_im][Cl]-treated aspen (**c**), and [C_4_C_1_im][MeCO_2_]-treated aspen (**d**)

The cell wall structure and spatial distribution of lignin in aspen and IL-pretreated aspen were compared by taking advantage of the autofluorescence of lignin (Fig. 1). The lignin in wood pretreated with [C_4_C_1_im][HSO_4_] (Fig. 1b) had a similar structure as that of untreated aspen (Fig. 1a), although the shape was slightly distorted, which may be the result of partial removal of hemicellulose. Wood treated with [C_4_C_1_im][Cl] exhibited a dilapidated lignin structure with only residual cell wall structure (Fig. 1c). As previous results show the strong effect of [C_4_C_1_im][Cl] on pure cellulosic substrates [13], the effect shown in Fig. 1c can be due to dissolution of cellulose resulting in a broken cell wall structure. Pretreatment with [C_4_C_1_im][MeCO_2_] resulted in a disordered lignin and no trace of the cell wall structure (Fig. 1d). This would reflect true homogeneous dissolution of the wood by [C_4_C_1_im][MeCO_2_] under the conditions studied, something that was not achieved with [C_4_C_1_im][Cl]. Thus, fluorescence microscopy based on lignin autofluorescence indicated increasing capacity to disorder the cell wall structure in the order [C_4_C_1_im][HSO_4_] < [C_4_C_1_im][Cl] < [C_4_C_1_im][MeCO_2_].

In additional sets of experiments, the structural effects of the IL treatment were analyzed using FTIR (Fourier-Transform Infra-Red) spectroscopy for estimation of crystallinity and SEM (Scanning Electron Microscopy) for analysis of surface structure. Analysis of the TCI (Total Crystalline Index) of non-torrefied aspen and spruce using FTIR indicated lower crystallinity after pretreatment with [C_4_C_1_im][MeCO_2_] (Additional file 1: Table S2). While effects of torrefaction or pretreatment with [C_4_C_1_im][HSO_4_] and [C_4_C_1_im][Cl] were difficult to discern (Additional file 1: Figures S1 and S2), SEM images of aspenwood pretreated with [C_4_C_1_im][MeCO_2_] showed a clearly disordered structure (Additional file 1: Figure S2). These results agree well with the observations made by fluorescence microscopy.

### Enzymatic saccharification

**Fig. 2:**
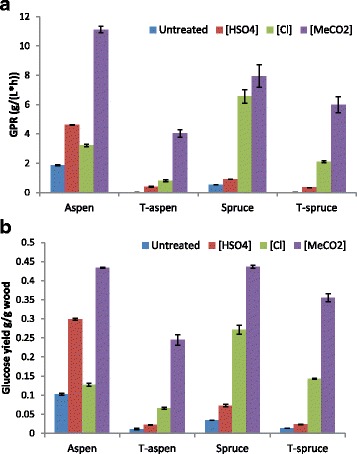
Enzymatic saccharification of non-pretreated and ionic-liquid pretreated non-torrefied and torrefied aspen and spruce. Data based on triplicate reactions: (**a**) Glucose Production Rate (GPR) after 2 h of enzymatic hydrolysis, and (**b**) glucose yield after 72 h of enzymatic hydrolysis

After the IL pretreatment, the precipitated material was hydrolyzed enzymatically and non-pretreated samples were used as controls. The saccharification results included the GPR and the glucose yields, which are shown in Fig. 2.

As expected, the hardwood (aspen) showed higher GPR value and glucose yield [1.9 g L^−1^ h^−1^ and 0.10 g g^−1^ wood, equal to 20% glucan conversion] than the softwood (spruce) [0.52 g L^−1^ h^−1^ and 0.034 g g^−1^ wood, equal to 7.0% glucan conversion]. Torrefied aspen and spruce were more recalcitrant than untreated wood, with undetectable GPR values and low glucose yields (0.011 g g^−1^ for T-aspen and 0.013 g g^−1^ for T-spruce, which correspond to 3.1% and 2.9% glucan conversion, respectively).

After pretreatment with ILs, significantly higher GPR values and glucose yields were achieved for all woody materials. Although acquired at different time points and analyzed with different methods, GPR values and glucose yields point in the same direction (Fig. 2).

For T-aspen, spruce, and T-spruce, both GPR values and glucose yields increased in the order: untreated < [C_4_C_1_im][HSO_4_] < [C_4_C_1_im][Cl] < [C_4_C_1_im][MeCO_2_] (Fig. 2). However, for aspen both GPR values and glucose yields instead followed the order: untreated < [C_4_C_1_im][Cl] < [C_4_C_1_im][HSO_4_] < [C_4_C_1_im][MeCO_2_].

The trends for non-torrefied aspen and spruce followed those observed by Gräsvik et al. [13] in the sense that [C_4_C_1_im][HSO_4_] worked better for aspen, whereas [C_4_C_1_im][Cl] worked better for spruce (Fig. 2). However, the torrefaction changed that pattern so that T-aspen became similar to spruce and T-spruce. The change can be attributed to the decreased xylan content caused by the torrefaction and to the capacity of [C_4_C_1_im][HSO_4_] to hydrolyze xylan. Pretreatment with [C_4_C_1_im][HSO_4_] decreased the xylan content for T-aspen from 2.6% to 1.4%, but these small fractions were probably not significant. Among the ILs tested, [C_4_C_1_im][HSO_4_] also had the best capacity to remove lignin from both aspen and spruce. However, the results indicate that the removal of lignin to the degree observed in the experiments was of less importance with regard to recalcitrance than the removal of xylan. Even though pretreatment of aspen with [C_4_C_1_im][HSO_4_] removed more hemicelluloses and lignin than the other ILs, the glucose yield (Fig. 2b) was only the second highest and amounted to 60% of the theoretical maximum. This can be compared with pretreatment of spruce with [C_4_C_1_im][Cl], which resulted in a glucose yield (Fig. 2b) that was 56% of the theoretical maximum.

Torrefaction always decreased the susceptibility to enzymatic saccharification (Fig. 2). As the torrefaction conditions used in this study hardly affected the glucan content, the decreased susceptibility of torrefied wood to enzymatic saccharification has to be attributed to increased recalcitrance. This can be explained by formation of pseudo-lignin (primarily from hemicelluloses) and its negative impact on enzymatic saccharification. Furthermore, the IL pretreatments were not as effective for mixtures of pseudo-lignin and real lignin in the same way as for lignin without any pseudo-lignin content. This can be seen in Table 2, as [C_4_C_1_im][HSO_4_] pretreatment removed 4.43 mg out of 9.79 mg lignin in aspen (no pseudo-lignin content), but only 1.05 out of 27.32 mg lignin in T-aspen (mainly pseudo-lignin). For removal of lignin IL pretreatment was slightly better for T-spruce (mainly real lignin) than for T-aspen (mainly pseudo-lignin) (Table 2), which again indicates better effect on real lignin than on pseudo-lignin. Despite the recalcitrance of the torrefied materials, pretreatment with [C_4_C_1_im][MeCO_2_] resulted in as much as 80% of the theoretical glucose yield (Fig. 2b) after enzymatic saccharification of T-spruce, which can be compared to 90% for spruce, 86% for aspen, and 69% for T-aspen.

## Discussion

The compositional analysis showed that the torrefied materials had a very high content of Klason lignin, which was attributed to formation of pseudo-lignin. Considering the conditions (285 °C for 16.5 min) that were used for torrefaction, hemicelluloses would be expected to be the main precursors of the pseudo-lignin. Cellulose and lignin are less sensitive to torrefaction than hemicelluloses [18, 19]. In a recent study of torrefaction of spruce wood, the impact of torrefaction on cellulose became apparent only when the severity was increased from 285 °C for 16.5 min or 310 °C for 8 min to 310 °C for 25 min [15]. Lignin was found to be even more resistant [15].

Brandt et al. [20] reported effective dissolution of hemicellulose and lignin by using [C_4_C_1_im][HSO_4_] with a water content of 20% at 120 °C for 22 h. While pretreatment of Miscanthus and willow resulted in high yields of glucose in subsequent enzymatic saccharification, the glucose yield from pretreated pine was, however, poor [20]. Karatzos et al. [8] compared the mass balance of sugarcane bagasse after treatment with [C_4_C_1_im][Cl], 1-ethyl-3-methylimidazolium chloride ([C_2_C_1_im][Cl]), and 1-ethyl-3-methylimidazolium acetate ([C_2_C_1_im][MeO_2_]). They reported preferential removal of lignin and acetyl by [C_2_C_1_im][MeO_2_], which also was found to be most suitable for biomass treatment [8].

Our investigation shows that although mass recovery decreased in the order [C_4_C_1_im][Cl] > [C_4_C_1_im][MeCO_2_] > [C_4_C_1_im][HSO_4_] as a result of partial removal of hemicelluloses and lignin, that order did not reflect the improvement of enzymatic saccharification, which was always best after pretreatment with [C_4_C_1_im][MeCO_2_]. Whereas the especially good effect of [C_4_C_1_im][HSO_4_] on saccharification of non-torrefied aspen can be explained by relatively efficient removal of hemicelluloses (especially xylan), analysis of the chemical composition of pretreated material could not explain the different effects of the ILs on non-torrefied spruce or on torrefied aspen and spruce. Fluorescence microscopy based on the autofluorescence of lignin was useful to evaluate the capacity of different ILs to introduce disorder in the cell wall structure. Introduction of disorder in the cell wall structure emerges as a more efficient way to improve enzymatic saccharification than partial removal of lignin and hemicelluloses. ILs that work by introducing disorder rather than by degrading the cell wall constituents would have an added advantage with regard to high mass recovery after addition of the anti-solvent.

## Conclusions

Analyses of chemical composition and mass balance combined with fluorescence microscopy were useful tools to elucidate different effects of anionic constituents of ILs on non-torrefied and torrefied aspen and spruce. The acidity of [HSO_4_]^−^ caused extensive hydrolysis of xylan and thus facilitated pretreatment of xylan-rich aspenwood. This was especially clear from analysis of torrefied samples, as torrefaction decreased the hemicellulose content and increased the Klason lignin content, which decreased the efficiency of [HSO_4_]^−^-based IL pretreatment of aspen in relation to ILs with other anions. Pretreatment with [C_4_C_1_im][MeCO_2_] resulted in efficient glucan-to-glucose bioconversion, even for a substrate as challenging as torrefied softwood. Taken together with the many advantages of torrefaction with regard to handling of lignocellulosic biomass [17, 18] this finding encourages further research on bioconversion of torrefied biomass.

## Additional file

Additional file 1: Table S1. By-products from the compositional analysis of the carbohydrate content. **Table S2.** Estimation of crystallinity using FTIR. **Figure S1.** SEM images of untreated and torrefied wood of aspen and spruce. **Figure S2.** SEM images of untreated and IL-pretreated aspenwood. (DOCX 2756 kb)
