# Supplementary material for: Comparison of [HSO4]−, [Cl]− and [MeCO2]− as anions in pretreatment of aspen and spruce with imidazolium-based ionic liquids
Source: BMC Biotechnol. 2017 Nov 15;17:82. doi: 10.1186/s12896-017-0403-0 (PMC5688671; doi:10.1186/s12896-017-0403-0)
Supplement: Additional file 1: Table S1. — By-products from the compositional analysis of the carbohydrate content. Table S2. Estimation of crystallinity using FTIR. Figure S1. SEM images of untreated and torrefied wood of aspen and spruce. Figure S2. SEM images of untreated and IL-pretreated aspenwood. (DOCX 2756 kb) [file 12896_2017_403_MOESM1_ESM.docx]

Additional files to:

Comparison of [HSO_4_]^-^, [Cl]^-^ and [MeCO_2_]^-^ as anions in pretreatment of aspen and spruce with imidazolium-based ionic liquids

by

Zhao Wang, John Gräsvik, Leif J. Jönsson and Sandra Winestrand

CONTENTS

Additional file Table S1

Additional file Table S2

Additional file Fig. S1

Additional file Fig. S2

**Additional file Table S1.** By-products from the compositional analysis of the carbohydrate content. The table shows aliphatic carboxylic acids (formic acid and levulinic acid) and furan aldehydes (HMF, i.e. 5-hydroxymethylfurfural, and furfural).^a^

| **Samples** | **Formic acid**  **(%, w/w)** | **Levulinic acid**  **(%, w/w)** | **HMF**  **(%, w/w)** | **Furfural**  **(%, w/w)** |
| --- | --- | --- | --- | --- |
| Aspen (control) | 1.6±0.4 | 1.0±0.1 | 0.5±0.0 | 1.7±0.1 |
| [HSO_4_] Aspen | 1.6±0.2 | 1.1±0.1 | 0.6±0.0 | 1.0±0.1 |
| [Cl] Aspen | 2.7±0.2 | 1.1±0.2 | 0.4±0.0 | 1.8±0.0 |
| [MeCO_2_] Aspen | 2.0±0.2 | 1.0±0.1 | 0.5±0.0 | 1.4±0.0 |
| T-aspen (control) | 2.3±0.4 | 0.7±0.1 | 0.3±0.0 | 0.2±0.0 |
| [HSO_4_] T-aspen | 1.8±0.2 | ND^b^ | 0.3±0.0 | 0.1±0.0 |
| [Cl] T-aspen | 1.5±0.4 | ND | 0.3±0.0 | 0.2±0.0 |
| [MeCO_2_] T-aspen | 1.2±0.3 | ND | 0.2±0.0 | 0.2±0.0 |
| Spruce (control) | 1.3±0.1 | 0.9±0.2 | 0.5±0.0 | 0.6±0.0 |
| [HSO_4_] Spruce | 1.8±0.2 | 1.0±0.1 | 0.5±0.0 | 0.3±0.0 |
| [Cl] Spruce | 1.1±0.2 | 0.8±0.1 | 0.5±0.0 | 0.6±0.0 |
| [MeCO_2_] Spruce | 1.8±0.1 | 1.0±0.2 | 0.5±0.0 | 0.5±0.0 |
| T-spruce (control) | 1.2±0.1 | 0.6±0.1 | 0.4±0.0 | 0.2±0.0 |
| [HSO_4_] T-spruce | 1.1±0.1 | 0.7±0.1 | 0.4±0.0 | 0.1±0.0 |
| [Cl] T-spruce | 1.1±0.1 | 0.9±0.1 | 0.4±0.0 | 0.2±0.0 |
| [MeCO_2_] T-spruce | 1.6±0.1 | ND | 0.4±0.0 | 0.2±0.0 |

^a^Separation and quantitation were performed using HPLC. Analytical procedures are described in Section "HPLC analysis of furan aldehydes and carboxylic acids".

^b^ND: not detectable.

**Additional file Table S2.** Estimation of crystallinity using FTIR. The table shows the Total Crystalline Index (TCI), i.e. the infrared crystallinity ratio (1372 cm^-1^/2900 cm^-1^) according to Nelson and O’Connor (1964).^a,b^

| **Material** | **Untreated** | **[C_4_C_1_im][HSO_4_]** | **[C_4_C_1_im][Cl]** | **[C_4_C_1_im][MeCO_2_]** |
| --- | --- | --- | --- | --- |
| Aspen | 0.93 | 0.88 | 0.91 | 0.66 |
| Spruce | 0.85 | 0.80 | 0.88 | 0.75 |
| T-aspen | 1.16 | 1.17 | 1.14 | 1.07 |
| T-spruce | 1.02 | 0.93 | 0.88 | 0.84 |

^a^Nelson ML, O’Connor RT. Relations of certain infrared bands to cellulose crystallinity and crystal lattice type. Part I. Spectra of lattice types I, II, III and amorphous cellulose. J Appl Polym Sci. 1964;8:1311–1324.

^b^FTIR spectra were obtained on a Bruker IFS 66v/S FTIR spectrometer with a standard Deuterated Triglycine Sulfate detector, and fitted with a diffuse reflectance accessory (Bruker Corporation, Billerica, MA, USA). The analysis was performed at the Vibrational Spectroscopy Core Facility (ViSP) of the KBC Chemical-Biological Centre (Umeå, Sweden). Milled wood samples were ground together with potassium bromide (KBr, Spectrograde, Fisher Scientific, Waltham, MA, USA) and the potassium bromide was set as background. The background and the measurements spectra were recorded with 128 scans and 4 cm^-1^ resolution. Experiments were carried out in vacuum. The spectra were baseline-corrected and the intensity ratios of 1372 cm^-1^/2900 cm^-1^ were used to characterize the crystallinity of the wood samples.


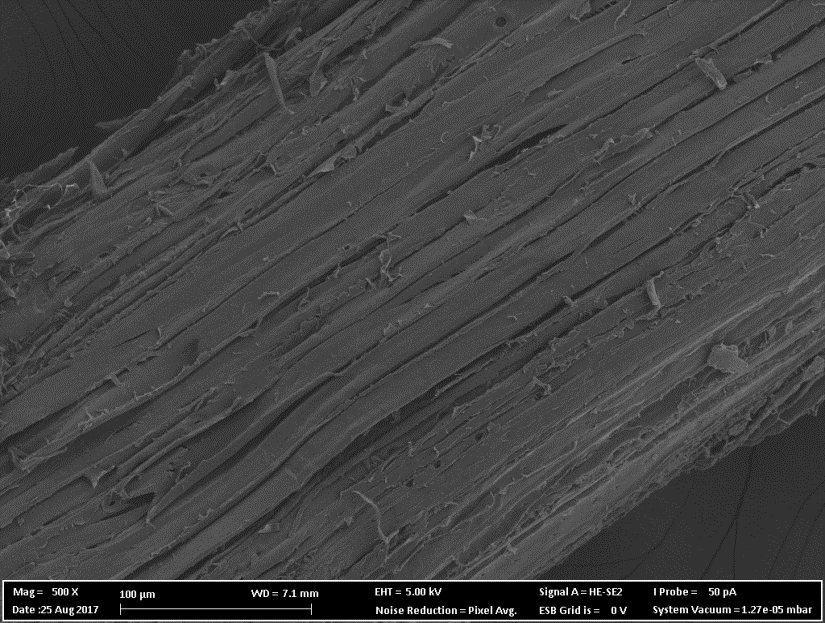

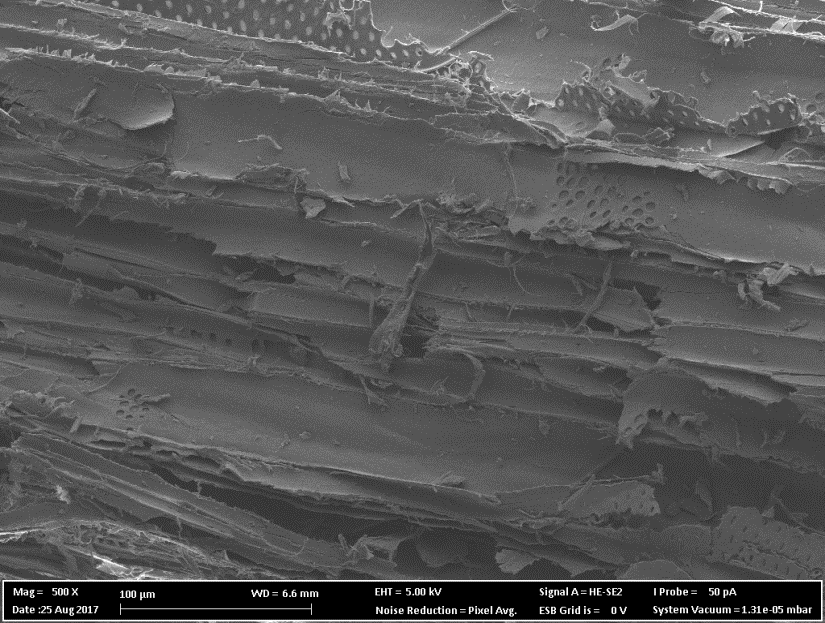

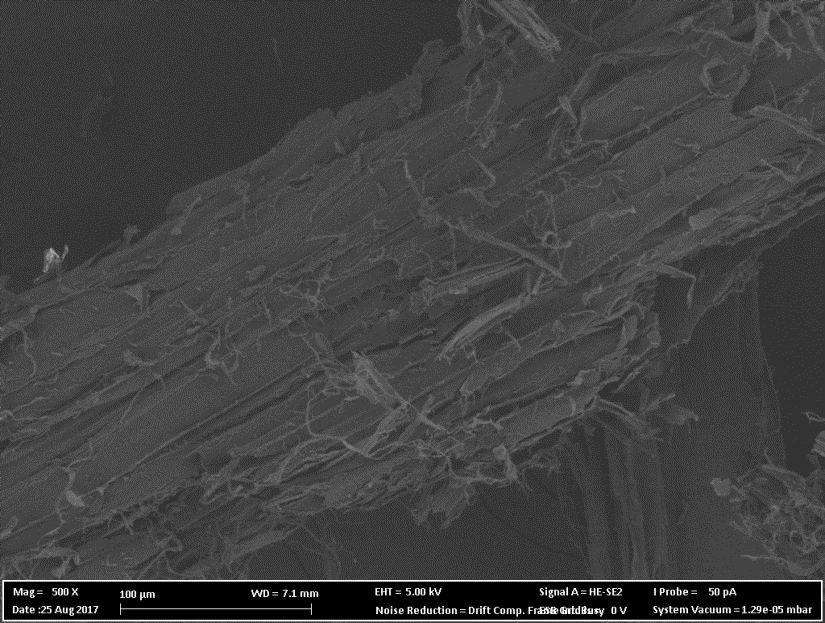

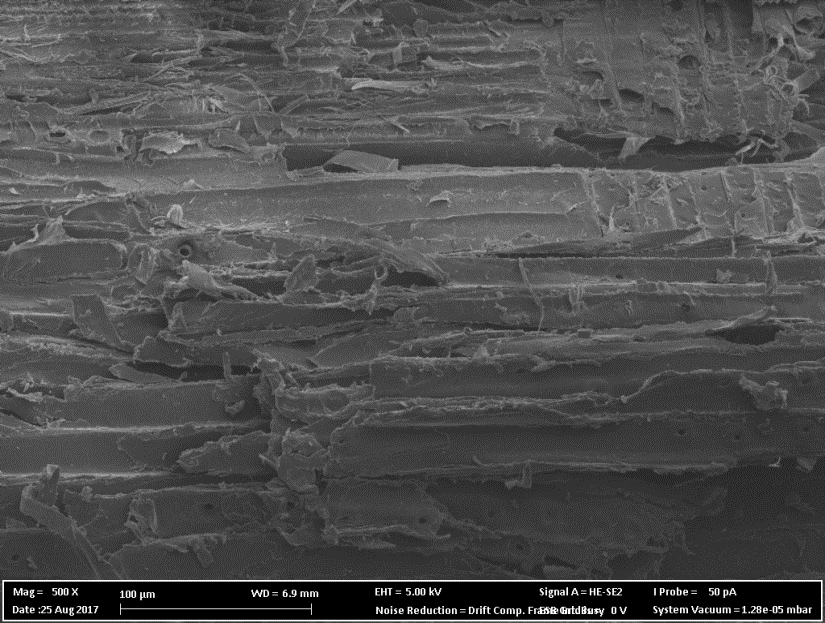


A

D

C

B

**Additional file Fig. S1.** SEM images of untreated aspen (A), torrefied aspen (B), untreated spruce (C), and torrefied spruce (D). Magnification: ×500. SEM analyses were carried out at the Umeå Core Facility Electron Microscopy (UCEM) of the KBC Chemical-Biological Centre (Umeå, Sweden). Samples were mounted on aluminum specimen stubs (Ted Pella, Redding, CA, USA) with carbon conductive tape (Ted Pella), and coated with Au/Pd (80:20). Observations were made by using a Field Emission Scanning Electron Microscope (Merlin FESEM, Carl Zeiss AG, Oberkochen, Germany) operated at 4 kV with images recorded digitally with the SmartSEM V.5.05 software.


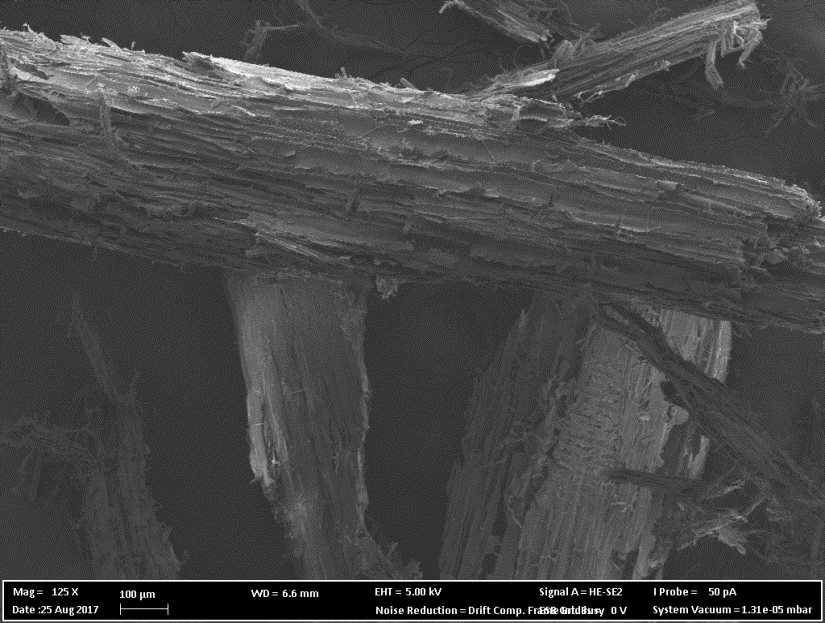

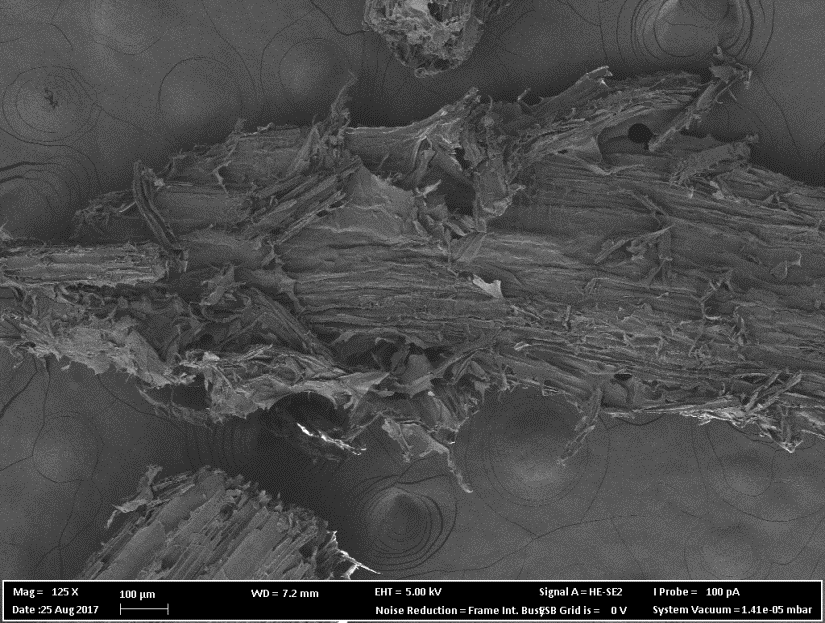

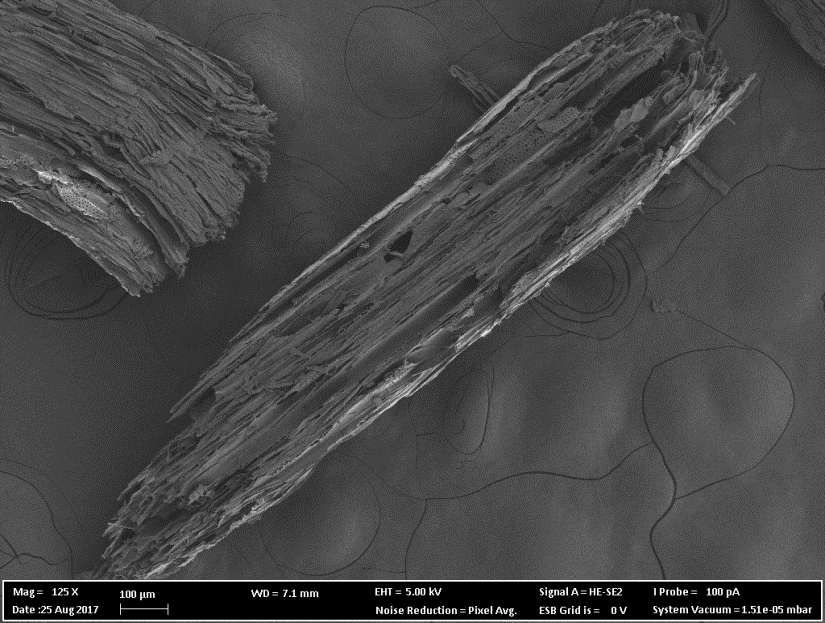

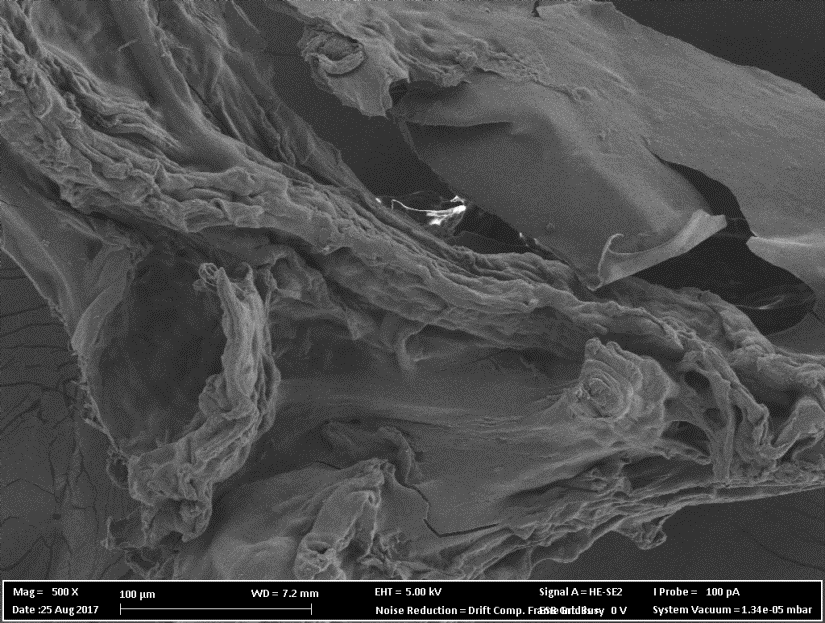


A

C

B

D

**Additional file Fig. S2.** SEM images of aspen (A), [C_4_C_1_im][HSO_4_]-pretreated aspen (B), [C_4_C_1_im][Cl]-pretreated aspen (C), and [C_4_C_1_im][MeCO_2_]-pretreated aspen (D). Magnifications: ×125 (A-C) and ×500 (D). Experimental procedures are indicated in the caption of Additional file Fig. S1.
